# Chromatin Memory in the Development of Human Cancers

**Published:** 2014-08-11

**Authors:** Yixin Yao, Thomas L Des Marais, Max Costa

**Affiliations:** 1 Department of Environmental Medicine New York University, New York, USA; 2 Department of Biochemistry and Molecular Pharmacology, New York University Langone Medical Center, Tuxedo, New York, USA

**Keywords:** Cancer epigenetics, Histone modifications, Methylation, Acetylation, Chromatin

## Abstract

Cancer is a complex disease with acquired genomic and epigenomic alterations that affect cell proliferation, viability and invasiveness. Almost all the epigenetic mechanisms including cytosine methylation and hydroxymethylation, chromatin remodeling and non-coding RNAs have been found associate with carcinogenesis and cancer specific expression profile. Altered histone modification as an epigenetic hallmark is frequently found in tumors. Understanding the epigenetic alterations induced by carcinogens or infectious agents may help us understand early epigenetic changes prior to the development of cancer. In this review, we focus on chromatin remodeling and the associated histone modifiers in the development of cancer; the application of these modifiers as a cancer therapy target in different clinical trial phases is also discussed.

## Introduction

Cancer is an extremely complex disease in terms of its etiology, clinical and molecular behavior. It is believed that tumor initiation and progression result from acquired genomic alteration within the originally normal cells, however, there’s increasing evidence supporting epigenetic impacts and epigenomics alterations in human cancer development without a change of DNA sequence [1,2]. Epigenetic impact includes changes in gene expression pattern derived by DNA methylation, histone modifications, ATP-dependent chromatin remodeling and non-coding RNA [3]. Nevertheless, an epigenetic change refers to heritable yet reversible alterations associated with gene regulations [4]. Within an individual, cells from different tissues are capable of maintaining their specific expression patterns despite of the fact that they share an exact same genome [5]. An “epigenetic restriction” was proposed to be the mechanism of how cells establish their identities, and therefore, it was even suggested that the study of epigenetics should be broadened to all changes in the regulation of gene activity and expression without change of DNA sequence [6]. “Cellular heritability” regarding epigenetic features that daughter cells inherit from mother cells is a major focus of epigenetic study of carcinogenesis and cancer therapy targets [7].

The initiation and development of cancer usually involve a nuclear reprogramming process to bring cells to their naive status and epithelial-mesenchymal transitions to facilitate metastasis, both of which exhibit a rebuilt of tumor cell specific epigenetic landscape [6,8]. This review focuses on chromatin remodeling and the associated histone modifiers in the development of cancer, the application of these modifiers as a cancer therapy target in different clinical trial phases is also discussed. For interests in other epigenetic aspects, extensive reviews can be found in area of LINE-1 methylation patterns in cancer cells [9], DNA methylation and the unique landscape of the DNA methylome in cancer [10,11], and alterations of non-coding RNAs in cancers [12].

## DNA Methylation and Cancer

### DNA methylation and demethylation

DNA methylation is a kind of modification that a methyl group is added covalently to 5-position of the cytosine [10]. The modified DNA bases act as regulatory marks that regulate gene expression in concert with their genomic location and density. In mammalian cells, the majority of 5-methylcytosine (5mC) is located within CG rich sequences, often occur in the promoter regions of genes and are called CpG islands. About 60% to 90% CpG islands are methylated and responsible for long term transcriptional silencing, such as genomic imprinting, X-chromosome inactivation, suppression of repetitive elements, as well as maintaining lineage specific gene silencing [13,14]. There are two basic mechanisms by which DNA methylation inhibits gene expression: direct blocking transcriptional activators from binding to cognate DNA sequences; and recruiting transcriptional repressors to silence gene expression through proteins that recognize methylated DNA [15]. Notably, while inversed correlation between gene promoter DNA methylation and gene transcription is wildly observed, gene body methylation which is called intragenic DNA methylation is more likely correlated to other functions such as modulate alternative promoter usage, production of intragenic non-coding RNA transcripts, cotranscriptional splicing, and transcription initiation or elongation [16]. Cancer cells have a unique DNA methylation profile and the DNA methylation alterations seen in cancer could due to both hyper- and hypo- methylation events [17,18]. These alterations are subject to environmental carcinogens’ influence and thereby a profile that resembles the methylome of a cancer cell could be induced [19,20].

DNA methyltransferases (DNMTs) catalyze the transfer of a methyl group from S-Adenosyl-L-methionine (SAM) to the carbon at position 5 of the cytosine. Newly synthesized DNA is methylated by DNMT1 by its binding to hemimethylated DNA during DNA replication and copying 5mC marks from the parental strand to the newly synthesized strand [10]. DNMT3A and DNMT3B are de novo methyltransferases establishing 5mC patterns in germ cells and developing embryos. 5mC is called the “fifth base” because of its importance in gene expression regulation [21]. The erasure of CpG methylation (5mC) is called demethylation. The demethylation process may be passive due to lack of maintenance methylation during several cycles of DNA replication, or as an active process without DNA replication [22]. The conversion of 5mC to 5-hydroxy-methylcytosine (5hmC) in mammalian cells by methylcytosine dioxygenase Ten-Eleven Translocation (TET) is thought to be an important procedure during active demethylation process [23]. 5hmc has been recognized as the “sixth base” in the genome because of its distinctive epigenetic role [24].

### DNA hypomethylation markers of human cancers

Cancer specific alterations of DNA methylome were previously considered to be predominantly hypomethylated DNA repeats and hypermethylated discrete gene regions. The development of high-resolution DNA methylome detection methods has revealed the complexity of cancer relavant DNA methylation markers [16]. A comparison of tissue specific and tumor specific DNA methylome [20] has led to the discovery of “CpG island shore” which denotes the sequence up to 2 kb distant from CpG island, adding another layer of complexity to human cancer DNA methylation markers. Surprisingly, cancer specific DNA methylation alteration at CpG islands was not as significant as previously speculated [25,26]. However, many traditionally acknowledged cancer DNA markers were still detected by new DNA methylome detection methods [20]. To distinguish causative epigenetic variations from the ones resulted from disease process is not easy, but is nevertheless crucial; this is because it will help to elucidate the functional role of the disease-associated variation and its potential utility in terms of diagnostics or therapeutics.

DNA repeats are often used as a surrogate for average genomic methylation measurement before high-resolution genome-wide analyses of DNA methylation techniques become available. It has been recognized that hypomethylation at highly repetitive sequences such as long interspersed nucleotide elements-1 (LINE-1) and short interspersed nucleotide elements (SINE) are epigenetic marks of cancer cells and tissue. LINE-1 is a kind of retrotransposon which is transcribed to RNA and processed during transposition. The processed RNA is reverse-transcribed by the LINE-1 encoded reverse transcriptase and the cDNA copy is inserted into a new chromosomal location [27]. LINE-1 is heavily methylated in majority cell types in mammals. Hypomethylation of LINE-1 occurs early during the process of carcinogenesis and the methylation level is usually further decreased in more advanced cancers [28]. Another kind of repetitive sequence, the Alu family, as the most abundant SINE; has also been found to be hypomethylated in breast cancer, colorectal cancer, etc [29–31]. The hypomethylation induces transcriptional activation of these sequences, which contributes to genomic instability and facilitates tumor progression; therefore, the methylation of CpG dinucleotides in repetitive sequences hosts defense against retrotransposon activation [32].

In intragenic region, cancer specific DNA methylation alterations have been found in both repeated and unique sequences including exonic and intronic sequences, CpG islands, CpG island shores, insulators, intragenic ncRNA sequences, and 3’ terminal regions [32–35].

Although “sixth base” 5hmC had previously been observed in mammalian genomes, it did not attract attention until the discovery of TET enzymes due to their capability of active generation of this base and thereby active demethylation of 5mC [36]. Down regulation of TET function has been observed in human breast, liver, lung, pancreatic and prostate cancers. Despite level variations of 5mC in various cancers, frequent TET mutational inactivation has been reported to associate with decreased 5hmC levels [37–39]; therefore, 5hmC has been proposed as a new epigenetic marker for human cancers.

### Biological impact of DNA hypomethylation in cancer development

While ageing and cancer seem often to be woven by similar complex molecular threads, an age-dependent decrease of global methylation has been observed both in normal tissues and in tumors [40]. Furthermore, hypomethylation of various repetitive sequences have been found associated with numerous carcinogenic exposures such as cigarette smoking, oxidative stress, etc. A significant positive correlation between LINE-1 hypomethylation and oxidative stress has been found not only in cancer patients but also in healthy individuals [41]. The consequences of hypomethylation throughout genomic repetitive sequences are genomic instability and alteration of gene expression, which will likely contribute to tumor heterogeneity and facilitate the survival of cancer cells in different environments.

LINE-1 methylation levels diminish early and progressively and correlate with tumor progression and prognosis. However, the correlation between methylation status of LINE-1 and cancers are not a one way street; for example, the hypomethylation of LINE-1 significantly increases the risk for head and neck cancers while LINE-1 methylation levels slightly increased with higher pack-years of smoking in blood samples of head and neck cancer patients [42]. Nevertheless, many LINE-1s have a role in gene expression regulation, and this control is regulated via the methylation at their 5’UTR [43]. Therefore, studies of location dependent and cell type dependent LINE-1 methylation pattern are needed to help understand its epigenetic impact on human cancer initiation and progession. The detailed LINE-1 methylation patterns in cancer cells have been reviewed comprehensively [29].

Intragenic DNA hypomethylation can also modulate the amount and type of RNA transcripts and thereby contribute to tumor formation and progression. Indeed, three genes whose expression has been reported to be altered in certain cancers were studied for their intragenic DNA methylation levels and interesting findings have been reported [16]. TGFB2 has an intronic Alu repeat that was hypomethylated in some cancer cell lines when compared to various normal tissues. PRDM16 exhibited gene body CpG island hypomethylation in an exon in some of the cancer cell lines. Last but not the least, NOTCH2 also showed gene body hypomethylation in several cancer cell lines, neither in a CpG island nor a DNA repeat. Notably, some of the cancer cell lines with TGFB2 or PRDM16 gene hypomethylation also displayed cancer cell linked promoter hypermethylation [16].

Loss of 5hmC has also been found in some human cancers. For example, melanoma showed significant decrease of 5hmc peaks at either promoters or gene bodies when compared to benign nevi or normal melanocytes, indicating it is a genome-wide event during melanoma progression [44]. Brain is one of the organs that with 5hmC presence at high levels. In terms of cancers developed in the central nervous system, it is observed that 5hmC levels decreased by clinical grades of the tumors; meaning high numbers of 5hmC positive cells in WHO grade I gliomas, fewer in grades II and III, and the least number of 5hmC positive cells in grade IV gliomas [24,45].

With increasing interest in the epigenetic impact of cancer development, some tumor suppressor genes that were traditionally thought to be key players in cell proliferation signaling pathway have been found to play an epigenetic role in tumor progression( tumor suppressor adenomatous polyposis coli (APC) )[46,47]. APC-deficient zebra fish embryos exhibited upregulated of DNA demethylase components, including cytidine deaminases Aid and Apobec2a, thymine glycosylase Mbd4, and DNA repair protein Gadd45a. Moreover, human colon adenoma with germ line APC mutations also showed reduced DNA methylation and upregulated Aid, Mbd4, and Gadd45a. The alterations were shown to be consequences of loss of retinoic acid production; for supplement with all-trans retinoic acid precluded the upregulation of Aid, Mbd4, and Gadd45a; and vice versa [46] .

### DNA hypermethylation markers of discrete genes in human cancers

Hypermethylation in tumor suppressor genes thereby silencing the genes by either physically inhibiting the binding of transcription factors, or by recruiting proteins that have transcription repressive properties has been reported in various human cancers. Many investigations have found cancer associated genes including cyclin-dependent kinase inhibitor 2A (p16), O6-methylguanine-DNA methyltransferase (MGMT), xeroderma pigmentosum group C (XPC), MutL homolog 1 (MLH1), breast cancer 1 and 2 ( BRCA1 and BRCA2 ), death associated protein kinase 1 (DAPK1), retinoic acid receptor β (RARβ), E-CADHERIN, CYCLIN A1, p14, p15, p73, RAS association domain family (RASSF1A) and APC genes are hypermethylated in cancers [48–51]. To support this notion, a broad set of carcinogenic exposures have been found associated with hypermethylation of these genes. RASSF1A methylation was significantly associated with increased asbestos body count [52]. Cigarette smoking has been identified as a risk factor associated with hypermethylation of p16, RASSF1A, RARβ, CDH13, MGMT and GSTP1, APC, and DNMT1 [53–55].

Although the link between gene promoter methylation and heritable transcriptional suppression is well recognized, the function of intragenic DNA methylation cannot be overlooked in terms of its role in cancer development. Methylation at the 5′ end of genes was found associated with transcriptional silencing whereas methylation in the more downstream portions of the gene body was not [56]. In breast cancer cells, hypermethylation of the second exon of antiapoptotic factor BCL-2 has been found associated with its diminished expression [57]. The second exon of BCL-2 contains a CpG island and an ER-binding site, which helped to explain why the endocrine resistant breast cancer cells exhibit increased sensitivity to cytotoxic chemotherapy agents such as paclitaxel [57].

### Biological impact of hypermethylation in human cancers

DNA hypermethylation may play a role in the etiology and pathogenesis of human cancer. The epigenetic silencing of MGMT leads to a greater mutation rate in K-RAS and p53 genes in human colorectal cancers [58,59]. Likewise, human papillary thyroid cancer samples with preferential hypermethylation of six genes (HIST1H3J, POU4F2, SHOX2, PHKG2, TLX3, and HOXA7) were significantly associated with mutation of BRAF/RAS oncogene [60]. Promoter hypermethylation induced inactivation of BRCA1 and MLH1 results in increased p53 gene mutation in human sporadic breast cancer [61,62] and microsatellite instability (MSI) in sporadic colorectal cancer [63] respectively. MSI in sporadic colorectal cancer are overwhelmingly due to epigenetic silencing of the MHL1 gene by hypermethylation of its promoter; this hypermethylation usually occurs in a background of widespread CpG island promoter methylation, also referred to as the CpG island methylator phenotype; which has also been found in gastric, lung, liver, ovarian, glioblastomas, endometrial and breast cancers [64].

The methylation status of repetitive sequences might affect some of the gene promoter hypermethylation found in human cancers. For instance, SINE B1 elements can influence the activity of proximal promoters and ultimately lead to epigenetic reprogramming [65]. Research results indicated that not all genes are equally sensitive to repression by retrotransposons, as MLH1 promoter activity was only moderately affected by B1 SINEs. It is speculated that the proximal promoter of MLH1 is protected from heterochromatinization by insulators; which helps to explain that MLH1 is methylated in a lower fraction of tumors compared with other genes such as p16 and DAPK1 in cancer tissue DNA methylation profiling [65].

An analysis of human genome SNP density to investigate the relationship between recent mutations and the methylation level in the human population has found that the unmethylated CpGs had a lower mutation rate (1.08%) when compared with methylated CpGs (3.55%) [66]. The underlying mechanisms include lower repair efficiency, higher rate of spontaneous hydrolytic deamination [67]. The major breakpoint region of bcl-2 found in follicular lymphoma patients are usually centered by the CpG sites [68]. A study using minichromosome system found that CpG sites must be methylated in order to be a focus of breakage and cytidine deaminases AID is also required for the CpG-focused breakage [69].

### Chromatin Memory: The Heritability of Chromatin Structure

**Figure 1:**
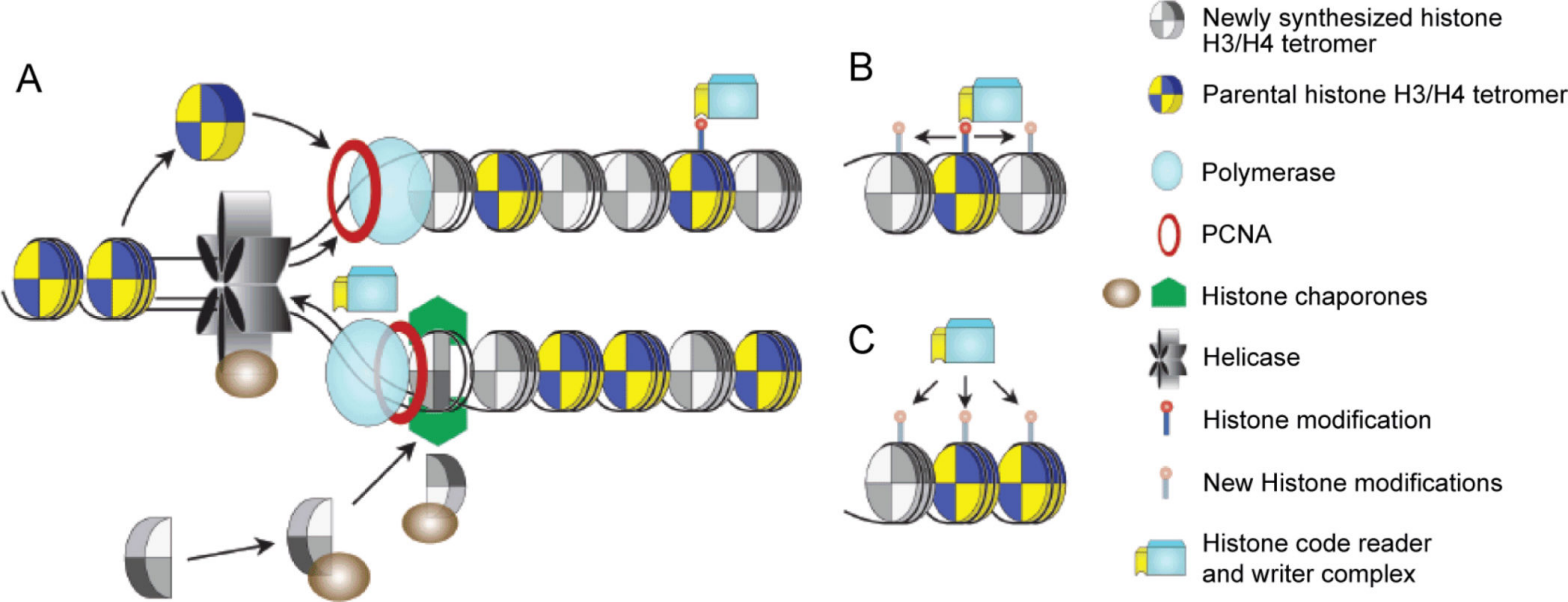
Proteins involved in chromatin memory. (A) Coupled nucleosome re-assembly at replication fork facilitated by histone chaperones and histone modifiers, namely the complex of histone reader and writer. (B) Possible mechanism of carrying over chromatin memory: direct copying of histone modifications onto newly synthesised H3/H4 tetromer using adjacent, parental nucleosomes as a template. (C) Possible mechanism of carrying over chromatin memory: the histone modifiers stay in close proximity of the replication fork to catalyze new histone marks in the absence of parental modifications.

Nucleosome, as the subunit of chromatin, is a complex of an octamer with two each of histone H2A, H2B, H3 and H4 wrapped by 147bp DNA [70]. Histone tails are subject to multiple post-translational modifications such as phosphorylation, methylation, acetylation, and ubiquitination. It has been suggested that the combination of these distinct covalent modifications of histones constitutes to the "histone code" and a variety of cellular processes are regulated according to the “code” [71]. The “histone code” cooperates with other epigenetic factors including DNA methylation and non-coding RNAs to modulate DNA accessibility and thereby change the gene expression pattern. Modifications that represent an active transcription include acetylation of H3 and H4, and di- or tri-methylation of H3 at lysine 4 (H3K4me2 or me3). On the contrary, methylation at H3K9 and H3K27 represents an inactivation of transcription [72]. Histones have to be removed and reused from the replicating DNA while genetic and epigenetic stability is ensured to transmit information at the replication fork, this process is coordinated by histone chaperones (Figure 1A) [73].

The inheritance of chromatin structure will be referred as “chromatin memory” from now on. Nucleosome re-assembly has proved to be important to couple DNA replication (disassemble before DNA replication and re-assemble after DNA replication) [74]. Histone chaperones are crucial mediators of nucleosome assembly and disassembly (Figure 1A) [74]. Cells lack of chromatin assembly factors showed impaired and/or stalled DNA replication [75,76]. In yeast, acetylated histones and histone chaperones have been found indispensable to faithful DNA replication [77,78]. Loss of H3K56 acetylation or complete loss of histone chaperones CAF1 (histone chaperone chromatin assembly factor 1) and Rtt106 (Regulator of Ty1 transposition protein 106, functionally redundant with CAF1) leads to a similar loss of replication intermediates which disrupt the genetic stability at replication fork [79].

We summarized two possible mechanisms of carrying over chromatin memory in Figure 1. One is direct copying of histone modifications onto newly synthesized H3/H4 tetromer using adjacent parental nucleosomes as a template (Figure 1B). Supporting evidence of this mechanism is often found in heterochromatinization process and the memory of heterochromatin. For example, heterochromatin protein HP1 is essential for heterochromatin formation [80], which can recognize H3K9me2/3 and in turn recruit the H3K9 specific histone methyltransferases SUV39H1/2 (Suppressor of variegation 3–9 homolog 1/2) and SETDB1 (SET domain, bifurcated 1) [81,82]. This association allows cells to copy modification from an adjacent nucleosome and helps explain the spreading of heterochromatic domains.

A recent study suggests that methylation of histones such as H3K4me3 and H3K27me3 may not be essential for DNA replication in very early drosophila embryos [83]. No methylated H3 were found to be associated with the newly replicated bulk DNA. On the other hand, epigenetic proteins, the Trithorax and Polycomb groups Trx, Pc, and E(z) are stable to DNA replication fork and are constitutively associated with nascent DNA instead of methylated histones through the S phase [83], indicating it’s the histone modifiers carrying over the memory of histone marks in the absence of parental modifications (Figure 1C). Consistent with this, the association of PCNA with heterochromatin formation components as well as histone chaperones have been reported [84,85], where they can modify newly loaded histones (Figure 1A).

The above models may be an oversimplification of the mechanism underlying chromatin memory. Intriguing enough, the H3K9me3 is inducible at OCT4 locus by a chemical recruitment of HP1, and the induced H3K9me3 is heritable even after several generations without the stimulation [86]. Unlike H3K9me3, DNA methylation was only slightly increased when transcription at the locus was fully repressed, but the promoter methylation continued to gradually increase and was significantly higher after a much longer period of time [86]. The stability of H3K9 methylation differs between cell types and varies in the context of transcription activity and DNA methylation level. H3K9me3 is retainable at low levels of promoter methylation and also after 5azaC treatment, but to retain H3K9me3 in the presence of a potent transcriptional activator, high levels of DNA methylation is needed to enhance heterochromatin stability in these cells [86]. This in vivo study has added a layer of complexity to the model of heritability of chromatin memory.

### The importance of chromatin remodeling

In eukaryotic cells, chromatin structures are dynamic and need to be constantly altered to accommodate DNA replication, gene transcription and stress responses. Alterations in the interaction between DNA and histones, together with the recruitment of nuclear proteins, cause changes in the chromatin structure, a process which is commonly referred to as chromatin remodeling [87,88]. Chromatin structure regulates gene transcription through histone displacement, histone variant incorporation, post-translational modifications affecting chromosome condensation, chromosome territories, and DNA looping [72]. Histone modifications are associated with the site-specific recruitment of chromatin remodeling proteins to carry out downstream effect and to alter gene expression profiles. For example, the importance of one of these genes EZH2 (enhancer of zeste 2 polycomb repressive complex 2 subunit) is evidenced by the lethality and embryonic growth arrest when lack of its function [89,90]. These impacts are consequences result from histone code readers interpreting specific combination of histone modifications through binding domains recognizing histone codes [71,91,92]. A good example is how HP1 interprets a combination of histone modifications. HP1 specifically binds to methylated H3K9 while phosphorylation at adjacent serine can displace HP1 from methylated H3K9 [93]. The adjacent modifications serve as a combination that can be recognized by “readers”; and functional outcomes are usually determined by the crosstalk of histone modifications and “readers”.

The chromatin remodeling has recently been found modulated by OGlcNAcylation, a kind of post translational modification with addition of β-D-N-acetylglucosamine to serine or threonine residues of nuclear and cytoplasmic proteins [94]. Most of the OGlcNAcylation sites on core histones have been found subject to phosphorylation, and hence OGlcNAcylation of these sites usually appear exclusively to the phosphorylation of these sites and increased H3 O-GlcNAcylation reduces phosphorylation and delays mitosis entry. [95] OGlcNAcylation appears to be wide spread on nuclear and cytoplasmic proteins and hence influencing chromatin remodeling and the crosstalk of histone and DNA modifiers at multiple layers. For instance, TET proteins interact with and target O-GlcNAc-transferase (OGT) to chromatin [96] and facilitate OGT interaction with chromatin and O-GlcNAcylation on H2A, H2B, H3, and H4 [97]. On the other hand, OGT associates and catalyzes the O-GlcNAcylation of all three TET proteins, but preferentially affects TET3 subcellular localization in an O-GlcNAc transferase activity-dependent manner [98], which will presumably affect DNA 5hmC status.

A specialized group of protein modules termed as plant homeo domain (PHD) finger has proven its importance of histone code readers in human cancer development [99]. Some PHD protein specifically recognizes tri- and di-methylated H3K4 (H3K4me3/2), with H3K4me3 as the preferred binding partner [100]. Others recognize unmodified histone residues such as H3R2 [101]. The ING (Inhibitor of Growth) family is one of the well-studied tumor suppressors with PHD finger at its C-terminal [102]. ING proteins are closely involved with different histone code “writers” such as histone acetyltransferases (HAT) and deacetylases (HDAC) in cells to further exert their influence on histone acetylation and chromatin remodeling, and thus, gene expression [102,103]. Other downstream effects of ING proteins include ensuring accurate DNA replication by regulating PCNA recruitment to the chromatin and replication speed to maintain genome stability [102], regulating DNA repair(“caretaker”) in response to cellular stress [103,104], regulating p53 post-translational modification(“gatekeeper”) in response to DNA damage [105,106], suppressing angiogenesis by inhibiting NF-кB [107]. Given their “caretaker” and “gatekeeper” tumor suppressing functions, it’s not surprising to find that ING family is lost or decreased in human tumors such as breast, ovarian, hepatocellular and lung cancers [108].

### Histone marks and their effects on chromatin structure in human cancer

Global and/or gene-specific histone covalent modifications change accompanied by alterations of enzymes associated with those marks is an epigenetic hallmark that is frequently found in tumor cells, similar alterations have also been found in carcinogen exposed cells [109].

Global loss of acetylation of histone H3 at lysine 9 (H3K9ac), H3K18ac, H4K12ac, H4K16ac, along with loss of trimethylation of histone H4 at lysine 20 (H4K20me3) and H3K4me2/me3 has been observed in various primary tumors and has been linked with tumor progression [110,111].

Trimethylation of histone H3 lysine 27 (H3K27me3) is usually a marker of transcriptional silencing. H3K27me3 seems to occur mutually exclusive to DNA methylation and promote de novo silencing of genes in different cancers [112,113] with a few exceptions [114]. Many genes that are silenced by H3K27me3 in embryonic stem cells are found silenced by DNA methylation in cancer cells, establishing an epigenetic switch from a differentiated state to a “stem cell like” signature of cancer cells. Increased H3K27me3 has been linked to poor prognosis in esophageal cancer cases, whereas in breast, prostate, ovarian and pancreatic cancers cases, patients exhibiting lower expression levels of H3K27me3 had significantly shorter overall survival time [115,116]. The answer to differentiation of H3K27me3 in different cancers might lie in its upstream pathways. It is reported that prostate cancers driven by oncogene MYC (v-myc avian myelocytomatosis viral oncogene homolog) in mice and human showed a reduced level of H3K27me3, and siRNA knockdown of MYC results in increased levels of H3K27me3 in prostate cancer cell lines [117]. In esophageal cancer patients, long non-coding RNA HOTAIR (HOX transcript antisense RNA) has been found up-regulated and critical for esophageal cancer cell metastasis in nude mice [118]. HOTAIR promote PRC2 recruitment to chromatin through its interaction with EZH2; thereby facilitate gene repression regulated by H3K27me3 [119]. Therefore, H3K27me3 might be an outcome of the dis-regulated upstream epigenetic machinery in different types of cancers.

Various combinations of histone modifications have been found in human cancers, and each combination reflects a disrupted balance between modifying and de-modifying enzymes, so-called code “writers” and “erasers”. Many of these enzymes are either cofactors or binding partners for transcription factors [120,121]. Therefore, a disregulation of histone modifiers could be a potential mechanism for tumor initiation. For example, (HATs) catalyze the transfer of an acetyl group from acetyl-CoA to the targeted lysine residues, and can further neutralize the positive charge of the lysines. The actions of HATs are countered by histone (HDACs) [122,123]. Structural confirmation induced by histone acetylations usually increases nucleosome mobility and DNA accessibility thereby facilitating transcription. Therefore, acetylation is associated with gene activation and deacetylation with gene repression [124,125]. Aberrant expression of HDAC family has frequently been shown to correlate with aggressive behavior of tumors and poor prognosis [126]. A subset of HDACs (HDAC1, 2, 3, 4, 5, and 11) was significantly up-regulated in liver cancer tissue in comparison to normal liver tissue. Furthermore, in hepatocellular carcinoma, HDAC3 and HDAC5 up-regulation was found correlated with their DNA copy number gains [127]. Nevertheless, HDAC inhibitors have shown their potential to modulate tumor suppressor and/or oncogene expressions, which make it increasingly tempting to consider HDAC inhibitors as one of the anti-cancer drugs [128–133].

Nevertheless, epigenetic states have been proven critical for genomic stability, too. Cancer mutation density over the genome is strikingly correlated with repressive histone mark H3K9me3 and H3K9me2 [90], indicating a closed chromatin structure is prone to mutation. The reverse correlation has been found with open chromatin marks including H3K4me3 and H3K9ac, which helps to explain why the global loss of these two marks is often observed in primary tumors [90,113,114,133]. This might be explained by the better accessibility of open chromatin structure, which provides DNA repair machinery a friendly working environment.

### Carcinogen induced alteration in chromatin confirmation

Similar alterations in chromatin structure have also been found inducible by carcinogens to resemble hallmarks of cancer. For example, global loss of H4K20me3 has been found inducible by hepatocarcinogens such as 1,3-butadiene and 2-acetylaminofluorene [134,136]. Potential carcinogen acrolein which has been found enriched in cigarette smoke and fumes of heated cooking oil, two known risk factors of lung cancer; is reported to induce acrolein-histone adduct as well as compromised H3 delivery and thereby impaired nucleosome assembly in cells exposed to acrolein [136].

In addition, histone mark variations in specific gene locations have also been reported in carcinogenesis. Metal and metalloid toxicants nickel (Ni), arsenic (As), and hexavalent chromium (CrVI) can cause global post-translational histone modification level change, as well as histone mark localization alteration [112]. For example, H3K9me2, a mark of transcriptional repression; and H3K4me3, a mark of transcriptional activation, were found increased in the promoter regions of several genes involved in the transcription of DNA into RNA and the synthesis of immune response cytokines [112]. Environmentally relevant solar ultraviolet A radiation (UVA) doses (1×20 or 4×5 J cm^−2^ per week) cause tumorigenic conversions of HaCaT skin keratinocytes after long-term exposure (10–15 weeks) [127]. The same treatments also induce reduction of the permissive mark H3K4me3 at P16INK4a , an important tumor suppressor gene whose silencing plays a pivotal role in photo carcinogenesis [138,139], accompanied by a substantial increase of its promoter DNA methylation, leading to a drastic decrease of the P16INK4a mRNA expression with 20-fold (for 10 weeks) and 40-fold (for 15 weeks) [140]. This epigenetic mechanism helps explain UV induced photo carcinogenesis.

Aside from in vitro studies, the research on occupationally exposed population with increased risk of lung cancer has indicated similar results. Occupational exposure to particulate matter and its metal components including nickel compounds through inhalation has been associated with lung and nasal cancers [141–143], while exposure to arsenic has been associated with skin, lung, bladder, kidney, and liver cancers [144,145]. Ex vivo exposure of Ni to peripheral blood mononuclear cells (PBMCs) from healthy subjects induced an increase in global levels of H3K4me3 and H3K9me2, which might well lead to a total of 1381 gene expression increase with a greater than 2-fold difference in expression in all treatments as compared to untreated control [145]. On the other hand, in vivo study of PBMCs of subjects with occupational exposure to high levels of nickel at a nickel refinery in China has found elevated global level of H3K4me3 (p=0.0004) and decreased global level of H3K9me2 (p=0.003) when compared to referent subjects [146]. These findings indicate environmental carcinogens might exert differential epigenetic effects under different circumstances, while some effect is reproducible in vivo and in vitro (elevated H3K4me3); others are not (alterations of H3K9me2).

In an independent in vivo study of workers in a steel plant, both H3K4me2 and H3K9ac were found increased in association with the years of employment of the study subjects in the steel plant [147]. H3K4me2 were found increased in association with nickel, arsenic, and iron exposure but not aluminum, manganese, zinc, lead exposure; H3K9ac was positively but not significantly associated with nickel and iron exposure [147].

### Histone associated protein deregulation in cancer and epigenetic therapies

Given the mounting evidence supporting the association of certain histone marks with carcinogenesis and high cancer risk occupational exposures, it’s not surprising to find histone modifications and their modifiers as valuable cancer therapy targets.

Enhancer of zeste (EZH)2, a methyltransferase component of polycomb repressive complex 2(PRC2) has been found deregulated in many sorts of cancers, including lymphoma [148,149], bladder [150–153], gastric [154,155], lung [156], breast cancers [157–159]. EZH2 catalyzes H3K27me3 in the presence of suppressor of zeste 12 (SUZ12) and embryonic ectoderm development (EED) which possess a carboxy-terminal domain specifically recognizes histone tails that carry trimethyl-lysine residues [160]. The trimethylation later serves as a docking site to recruit polycomb complex PRC1 followed by ubiquitination of H2AK119 to maintain the gene repression at these loci [160]. The deregulation of EZH2 found in cancers includes an over-expression of wild-type protein [151,161,162] and a gain-of-function mutation resulting in a switch from tyrosine to histidine at amino acid 641(Y641H); both of which lead to a hyper-trimethylation on H3K27me3 [163].

An inhibitor of EZH2, 3-Deazaneplanocin (DZNep), is hence proposed to be tested as a method of epigenetic cancer therapy (Table 1) [164]. DZNep down-regulates EZH2 protein and therefore the polycomb function through inhibiting S-adenosylhomocysteine hydrolase, which induces an accumulation of the enzyme substrate adenosylhomocysteine and in turn inhibits methyltransferases [165]. A study using four non-small cell lung cancer cell lines found DZNep treatments led to decreased cell proliferation and less anchorage independent growth [165]. An independent study revealed a treatment of DZNep combined with DNA methyl transferase inhibitors result in a decrease of H3K27me3 on MAGE (the melanoma antigen gene) family regulatory regions and subsequently increased their expressions. The increased expressions of MAGE sensitize the tumor cells and make them better targets for T cells [166].

While epigenetic therapy targeting EZH2 is still at its pre-clinical phase, other epigenetic therapies of different targets have been carried out in phase I/II trial and appeared encouraging. A phase I/II trial with combined epigenetic therapy with a DNA methyltransferase inhibitor azacitidine and a histone deacetylase inhibitor entinostat showed a favorable survival when comparing with existing therapeutic options (Table 1) [166]. Another phase I clinical trial in patients with newly diagnosed diffuse large B-cell lymphoma showed that combined treatment with the azacitidine and standard chemoimmunotherapy is feasible and that azacitidine treatment results in sensitization of lymphoma to chemotherapy in these patients (Table 1) [168].

## Conclusion remarks

The emerging fundamental roles of altered epigenetic machinery in cell transformation and carcinogenesis have implicated that medication targeting these players is a new frontier for drug discovery. So far, only a few compounds targeting histone modification enzymes are available for preclinical and clinical development due to their toxicity and limited knowledge. The epigenetic mechanisms for the involvement of various novel epigenetic pathways in cellular transformation and early carcinogenesis remain largely unexplored. Although the epigenetic chemo-preventive strategy study remain inconclusive, understanding the epigenetic mechanisms underlying the window of operational reversibility during early carcinogenesis induced by carcinogen or infectious agent may be helpful to design new strategy to revert or halt these early epigenetic changes prior to the development of cancer.

## Figures and Tables

**Table 1:** Epigenetic inhibitors and their targets

Inhibitor	Target	Stage	Cancer type
DZNep (3-Deazaneplanocinn A)	EZH2	preclinical	non-small cell lung cancer
azacitidine	DNMT	phase I clinical trial	diffuse large B-cell lymphoma
entinostat	HDAC	phase I/II clinical trial	non-small cell lung cancer
azacitidine	DNMT	phase I/II clinical trial	non-small cell lung cancer
